# The Effect of Age on Prostate Cancer Survival

**DOI:** 10.3390/cancers14174149

**Published:** 2022-08-27

**Authors:** Roderick Clark, Danny Vesprini, Steven A. Narod

**Affiliations:** 1 Division of Urology, Department of Surgery, University of Toronto, Toronto, ON M5G 2C4, Canada; 2 Women’s College Research Institute, Women’s College Hospital, Toronto, ON M5S 1B2, Canada; 3 Department of Radiation Oncology, University of Toronto, Toronto, ON M5T 1P5, Canada; 4 Dalla Lana School of Public Health, University of Toronto, Toronto, ON M5T 3M7, Canada

**Keywords:** prostate cancer, mortality, epidemiology

## Abstract

**Simple Summary:**

It is a commonly held belief that elderly men with prostate cancer are less likely to die of their cancer than are younger men because they have a higher risk of dying of another cause. This has impact on prostate screening policies and the decision to offer aggressive treatment. It is not clear to what extent the age of diagnosis and the current age impact on prostate cancer survival. We estimated prostate cancer survival rates and annual mortality rates according to age of diagnosis using data from the SEER program. We identified 116,796 prostate cancer patients diagnosed between 1992 and 1997 and followed them for 20 years. Among men diagnosed before age 70, 17% died of prostate cancer. Among men diagnosed after age 70, 21% died of prostate cancer. For men with low-grade cancers, the annual risk of dying of cancer rose continuously with time since diagnosis and peaked in men 85 years and older.

**Abstract:**

It is not clear to what extent the age of diagnosis and the attained age impact on cancer mortality rates in men with newly diagnosed prostate cancer. We estimated annual prostate cancer mortality rates and 20-year survival rates according to the age of diagnosis, race, grade and time since diagnosis using data from the Surveillance, Epidemiology and End-Results (SEER) program. We identified 116,796 prostate cancer patients diagnosed between 1992 and 1997 and followed them for 20 years. There were 21,896 deaths from prostate cancer. We calculated actuarial survival rates and annual prostate cancer mortality rates by age of diagnosis and by tumor grade. The risk of a man dying of prostate cancer was 17% for men diagnosed before age 70 and was 21% for those diagnosed after age 70. The mean annual prostate cancer mortality rate calculated over the 20-year period post-diagnosis was 1.5%. The annual rate increased from 0.9% for those diagnosed below age 60 to 2.1% for those diagnosed above age 70. For men with Gleason score ≥ 7 prostate cancer, the annual prostate cancer mortality rate peaked 2–3 years after diagnosis and then declined. For men diagnosed with Gleason score ≤ 6 prostate cancer, the annual prostate cancer mortality rate continued to rise 20 years after diagnosis and peaked after age 85. This suggests that high-grade prostate cancers are aggressive from the outset, but that low-grade prostate cancers may enter a state of dormancy and reactivate as the patient ages.

## 1. Introduction

Approximately one in eight men who are diagnosed with prostate cancer will ultimately die of it [1,2,3]; the majority of prostate cancer patients will die of another cause. The risk of a patient dying of prostate cancer varies according to clinical factors, which include tumour grade and stage, race and the age of diagnosis [4]. It is assumed that men above age 70 may not benefit from prostate cancer screening because, even if prostate cancer is detected, they are more likely to die of another cause (or may benefit less from treatment) than are younger men [5]. It is acknowledged that the risk of prostate cancer (incidence) increases with age [6], but there is less age-specific data on case-fatality [6,7,8,9]. It is not known to what extent the observed effect of increasing age on mortality reflects the intrinsic (pathologic and mutational) characteristics of the prostate cancer at diagnosis (cell autonomous), lack of effective treatment, or the waning resistance of the aging host (macro-environment) [9,10,11]. 

There are several methods of representing prostate cancer mortality. Actuarial survival curves generated by the Kaplan–Meier method are commonly used [12,13] but they may not adequately reflect changes in mortality rates with time since diagnosis. A second method is to estimate the ultimate probability of death. This is relevant to the patient (what are the chances I am going to die from my cancer?). A third method is to present the annual mortality rates from prostate cancer for each year post-diagnosis (what are the chances I will die from this cancer in any given year?) [14]. This method is helpful to illustrate changes in annual mortality rates over time and adjust for competing causes of death. By applying these different methods to a large cohort of prostate cancer patients, we can obtain a better appreciation of patterns of mortality from prostate cancer and how these patterns change with age. We can also speculate as to the relative contributions of the cancer cell and the host on the burden of mortality [11].

The objective of our study was to explore the impact of age of diagnosis and of current (attained) age on prostate cancer mortality for 25 years from diagnosis. We used a large US-population-based cohort of unselected prostate cancer patients diagnosed in a narrow five year time window and compared mortality patterns by age of diagnosis and by attained age. The SEER cohort is ideal because it is large and each patient has 20 years of follow-up from diagnosis [15]. 

## 2. Materials and Methods

### 2.1. Study Design, Data Source and Population

We performed a population-based, retrospective cohort study using data from the Surveillance, Epidemiology and End-Results (SEER) program. The SEER research database captures population-based data from cancer registries which cover roughly 34% of the US population. We used SEER*Stat statistical software, version 8.3.6 (National Cancer Institute, Bethesda, MD, USA) to conduct a case-listing session and retrieved all cases of men with primary invasive prostate cancer in the SEER 18 registries research database (November 2016 submission). All data was extracted using a SEER Stat case listing session [15]. Our inclusion criteria were men with American Joint Committee on Cancer (AJCC) prostate cancer diagnosed from 1992 to 1997. This study was exempted from review by the Women’s College Hospital research ethics board, because patient informed consent was not required. We adhered to the “Strengthening the Reporting of Observational Studies in Epidemiology Statement” guidelines for reporting observational studies [16]. 

For each case, we retrieved age of diagnosis and ethnic group. Prostate cancer characteristics included year of diagnosis, tumor size (T stage) and grade, nodal status (N stage), AJCC stage. Treatment details included the type of surgery received (radical prostatectomy versus other). Information on other therapies including radiotherapy was not available. Our primary outcome was death from prostate cancer. We extracted information on survival time from the variable “survival time months.” The SEER*Stat program estimates survival time by subtracting the date of diagnosis from the date of last contact (study cut-off). Data were analyzed using STATA (STATA, version 11.5, StataCorp LLC, College Station, TX, USA). Details on how the cause of death was determined are not provided in the SEER data set.

### 2.2. Outcome, Exposure and Covariates

Our primary exposures were age at diagnosis, tumour grade and ethnic group. We collected information on vital status, date of death, cause of death and months of survival. Our primary outcome was death from prostate cancer. For the purposes of this paper we use the term mortality instead of case-fatality.

### 2.3. Statistical Analysis

We measured survival in four ways: (1) the crude probability of dying from prostate cancer. (2) the annual mortality rate from prostate cancer by time since diagnosis and by attained age; (3) the 20-year actuarial cumulative likelihood of death from prostate cancer (4) the distribution of times from diagnosis to death from prostate cancer. 

The crude probability of dying of prostate cancer was the ratio of all men in the study who died of prostate cancer in the twenty-year follow up period and the number of men entered into the study.

The annual mortality rate was calculated for each year post-diagnosis. The annual mortality rate is the probability of dying in a given year, conditional on being alive at the beginning of the year. This is a percentage figure, based on the ratio of number of deaths from prostate cancer in a given year after diagnosis compared to the number of men who were alive at the beginning of the given year. For example, if there were 1000 patients alive at the beginning of year 3 and 20 died during year 3 then the annual mortality rate for year 3 was estimated to be 2%. We calculated this annual rate by years from the date of diagnosis and by the actual age of the patient. A patient contributed person years from the age of diagnosis until either death from prostate cancer, death from another cause or until 31 December 2017. To generate age-specific mortality rates, we summed the total person years at risk for each one-year age interval from 40 to 90. A patient contributed person years from the age of diagnosis until either death from prostate cancer, death from another cause or until 31 December 2017. Next, we counted the number of deaths from prostate cancer that took place within each one-year age interval. The age-specific annual mortality rate was the ratio of deaths from prostate cancer divided by the number of person years for each one-year interval from age 40 to age 80. 

The crude actuarial cumulative likelihood of death from prostate cancer at 20 years was calculated using the Kaplan-Meir method [10]. Patients were followed from the age of diagnosis to the age of death from prostate cancer, age of death from another cause or 31 December 2017.

The distribution of times from diagnosis to death is presented as a histogram for those men who died of prostate cancer. This was a crude description and was not adjusted for the size of the population at the beginning of the yearly interval. The histogram did not include men who were alive at the end of follow-up or who died of another cause. 

## 3. Results

Our study included 116,796 men diagnosed with prostate cancer between 1992 and 1997 (Table 1). Most patients self-reported as white (81.9%), followed by African American (12.5%) and other ethnicities (American Indian/Alaskan native, Asian; 5.4%). The majority (69.3%) were diagnosed with low grade (Gleason ≤ 6) prostate cancer. A small proportion (6.3%) were diagnosed with metastatic prostate cancer at diagnosis. 

The survival experience of the patients is summarized in Table 1. We present the crude number of deaths from prostate cancer, the annual prostate cancer-specific and all-cause mortality rates and the 10- and 20-year actuarial risks of prostate cancer death. We also present the proportion of deaths that occurred early after diagnosis (years 1 to 10) and those that occurred later on (years 10 to 20) (deaths occurring after 20 years are not shown).

### 3.1. Proportion Who Died

Of the 116,796 men in the study, 92,590 (79%) have died; 18.7% died of prostate cancer and 58.3% died of another cause. The median age of diagnosis was 70 years. The median age of death from prostate cancer was 79.5 years and the median age of death from other causes was 82.9 years. The probability of death from prostate cancer was 18% for white men and was 24% for African American men.

The crude probability of death from prostate cancer increased with age of diagnosis; of the men diagnosed before the age of 60, 15.4% died of prostate cancer, of the men diagnosed between age 60 and 70, 16.9% died of prostate cancer and of the men who were diagnosed after age 70, 21.4% died of prostate cancer (Table 1). 

Of the men who presented with non-metastatic prostate cancer of Gleason score ≤ 6, 10.6% died of prostate cancer and of the men who presented with non-metastatic prostate cancer of Gleason score ≥ 7, 27.7% died of prostate cancer. Forty-six percent of all the prostate cancer deaths occurred in men initially diagnosed with low grade disease (Gleason Score ≤ 6). Of the 7416 men who had metastatic disease at presentation, 65% died of prostate cancer. 

### 3.2. Annual Mortality Rates

**Figure 1:**
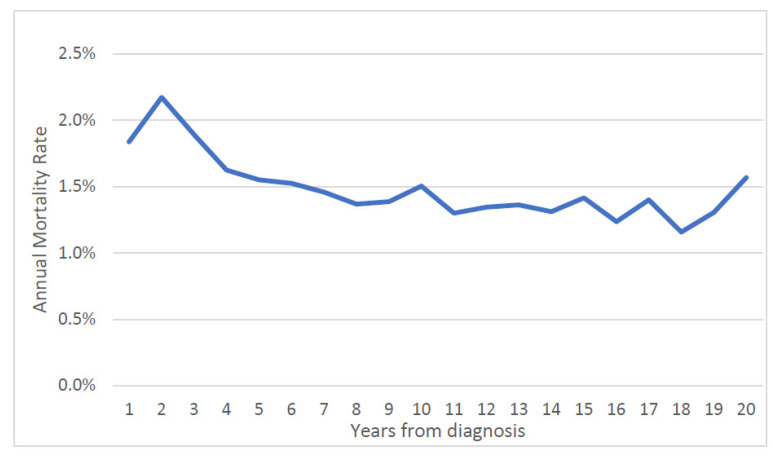
Annual prostate specific mortality rate by time from diagnosis (entire cohort).

**Figure 2:**
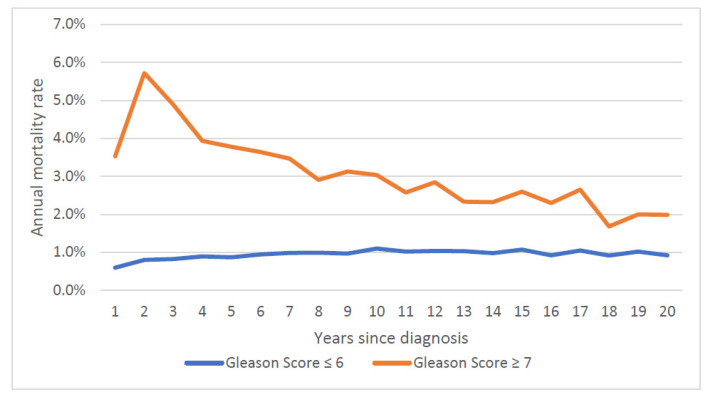
Annual prostate specific mortality rate, by Gleason score.

Over the first twenty years, the mean annual prostate cancer-specific mortality rate was 1.5% for the entire cohort (Figure 1) and was relatively steady. The average annual prostate-specific mortality was 1.9% for African American patients and was 1.4% percent for white patients. The annual prostate cancer-specific mortality rate was 0.9% for men initially diagnosed with Gleason score ≤ 6 cancer and was 3.1% for those diagnosed with Gleason score ≥ 7 cancer. The gap in the annual mortality rates for the two groups narrowed over the 20-year follow-up period (Figure 2). 

**Figure 3:**
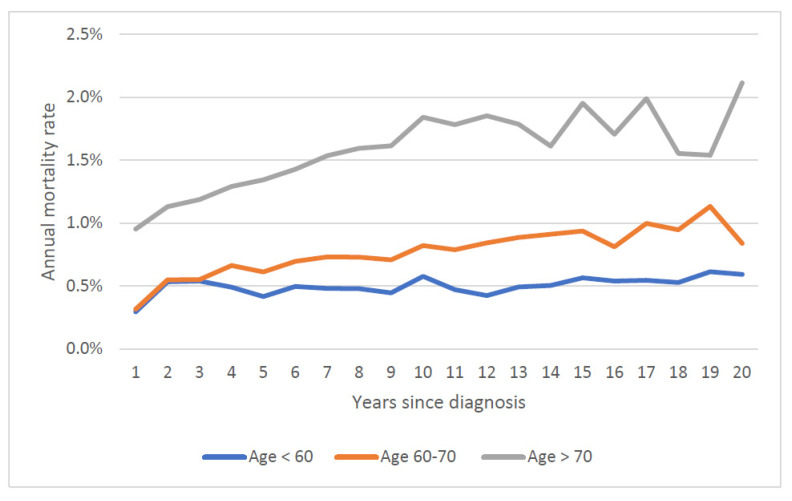
Annual prostate specific mortality rate by age of diagnosis (Gleason Score ≤ 6).

**Figure 4:**
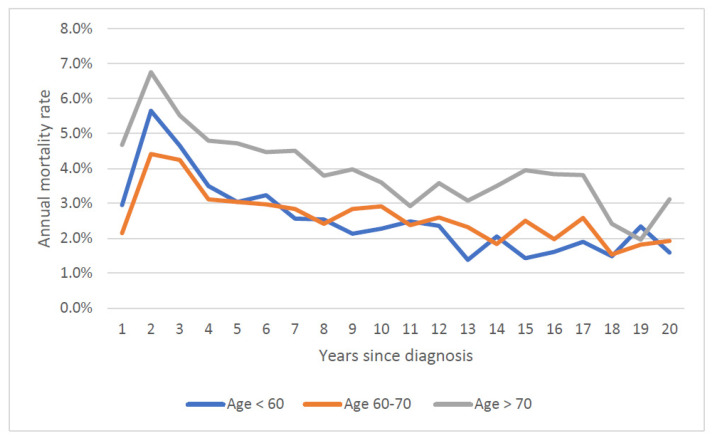
Annual prostate specific mortality rate by age of diagnosis (Gleason Score ≤ 7).

The mean annual prostate cancer specific mortality rate over 20 years increased with age at diagnosis; it was 0.9% for those diagnosed under 60, 1.2% for those diagnosed from age 60 to 70 and 2.1% for those diagnosed age 70 and above. Mortality rates stratified by both age at diagnosis and Gleason score are presented in Figure 3 and Figure 4. Within all age groups, the annual mortality rate for men with low-grade cancers increased with time from diagnosis (Figure 3). Within all age groups, the annual mortality rate for men with high-grade cancers declined with time from diagnosis (Figure 4). 

**Figure 5:**
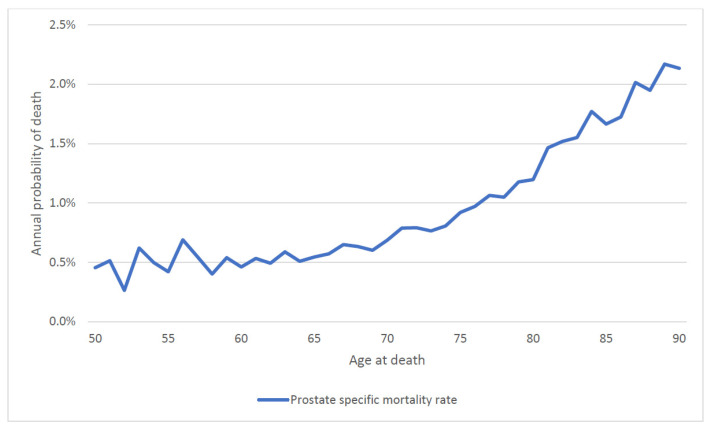
Annual prostate specific mortality by attained age (entire cohort).

**Figure 6:**
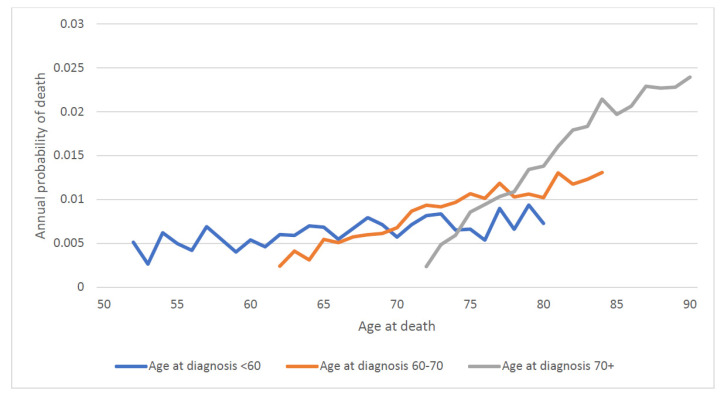
Annual prostate specific mortality by attained age and age of diagnosis.

We then calculated the annual mortality rate by attained age. For the entire patient cohort, the annual probability of dying of prostate cancer rose continuously with attained age (regardless of age of diagnosis) from 0.5% a year at age 50 to 2.0% a year at age 90 (Figure 5). To differentiate the effect of age of diagnosis vs. attained age on prostate cancer mortality, we stratified the cohort by age at diagnosis (Figure 6). For men diagnosed at age 60 and above, prostate cancer mortality increased sharply as they aged (Figure 6). 

For men with low-grade prostate cancer, the annual risk of dying was 0.4% per year between ages 50 and 60, was 0.6% per year between the ages of 60 and 75 and was 1.5% per year above age 75. The increase in the annual prostate mortality risk with age was largely independent of the age of diagnosis (the age-specific annual mortality risks were similar for men in each category of age at diagnosis). Among men with high-grade prostate cancers the annual risk of dying of prostate cancer was 3.7% per year between ages 50 and 60, was 3.0% per year between the ages of 60 and 75 and was 4.1% per year above age 75. The annual risk of dying of prostate cancer was highest after the men reached age 75.

### 3.3. Actuarial Survival Rates

The (traditional) Kaplan–Meier curves are presented in Figures S1–S4. We observed inferior survival for men diagnosed over 70 versus those diagnosed at a younger age, for African American men compared to white men and for men with Gleason scores 7 and above compared to 6 and below.

### 3.4. Time to Death

Among the 21,896 men who died of prostate cancer, the median time to death was 5.0 years. The median time to death was 7.5 years for those diagnosed under 60, was 7.8 years for those 60 to 70 and was 4.8 years for those 70 and above. Overall, 69.3% of the deaths occurred in the first ten years after diagnosis and 30.7% occurred after ten years. The distributions of time to death are presented by age of diagnosis (Figure S5) and by Gleason score (Figure S6). For patient subgroups with high mortality rates, the majority of deaths occurred in the first ten years (Table 1). For patient sub-groups with lower mortality rates, the majority of the deaths occurred after ten years. For example, among white men under age 60 with no metastases at diagnosis and low-grade disease, the median time to death from prostate cancer was 12.1 years. In contrast, for African American men over 70 with no metastases and high-grade disease, the median time to death was 4.7 years.

## 4. Discussion

In this large cohort study, we found the annual prostate-specific mortality rate increased with both age at diagnosis and with attained age, with the result that older men had relatively poor survival. Of the men diagnosed before age 60, 14.4% died of their prostate cancer, whereas of the men diagnosed after age 70, 21.1% died of their prostate cancer. This is despite the fact that men over age 70 had a shorter life expectancy than younger men and were more likely to die of another cause. For men with low-grade disease, those who lived into their eighties experienced a surging mortality rate from prostate cancer (Figure 5). Thus, it should not be presumed that older men are less likely to be impacted by a prostate cancer diagnosis than younger men because competing causes of death reduce their likelihood of dying of prostate cancer.

Several other studies have reported on long-term survival associated with prostate cancer according to age at diagnosis, but these have had smaller sample sizes and were based on groups of patients selected for treatment [7,8,9,10]. Here, we present crude survival rates and in many studies the crude effect of age on survival is masked by adjustment for covariates, such as grade, PSA, treatments received and comorbidity. It is ideal to first present the crude actuarial survival rates by age group and then to compare these with adjusted rates in order to better understand the features that contribute to the poor mortality experience of elderly men with prostate cancer, compared to younger men.

It is also necessary to follow prostate cancer patients for 20 years to appreciate long term patterns in mortality rates. In the present study, we divided the patient cohort by grade and this highlights the differences in mortality patterns between high grade and low grade cancers. In particular, the maximum mortality rate is not achieved until 20 years post-diagnosis for men with low grade cancer (Figure 3).

Bechis et al. reported on the impact of age of diagnosis on crude survival among 11,790 treated men in the CaPSURE database [7]. Older men were more likely to be diagnosed with high risk prostate cancer than younger men. In this study age of diagnosis was a crude (univariate) predictor of prostate cancer specific mortality, survival was poor for men diagnosed over 75. However, the age effect was not significant after controlling for risk category and treatment.

Using the SEER Registry, Bernard and colleagues showed an effect of increasing age on survival from patients diagnosed with metastatic prostate cancer [8]. The effect was most notable for men diagnosed at age 75 and older and was present after adjustment for treatment received.

Pettersson et al. conducted a nation-wide cohort study of 121,392 Swedish prostate cancer patients diagnosed from 1998 to 2012) [9] with results similar to ours. The five year mortality from prostate cancers rose from 7% at age 65 to 25% at age 80. They found old age to be a predictor of death among men treated with prostatectomy or followed with active surveillance, but not among those treated with radiotherapy. The authors conclude that lack of PSA screening in elderly men and the withholding of curative treatments were among the factors impacting upon their survival, but there was an inherent age effect as well.

Hall et al. explore the extent to which comorbidity explains the poor relative survival of elderly men with prostate cancer [10]. They conclude that comorbidity was one of the factors that was associated with poor survival in elderly men, but to a large extent those with serious comorbid conditions were less likely to be offered curative therapy.

It is not entirely clear why case-fatality increases with age of diagnosis. To our knowledge this phenomenon is unique to prostate cancer. It does not appear that older men present with more aggressive cancer. Among men diagnosed under age 70, 59% had Gleason grade 6 disease and 3.1% had metastatic disease at presentation. Among men diagnosed at or over age 70, 55% had Gleason grade 6 disease and 4.1% had metastatic disease at presentation. It may be that older men are less likely to receive curative treatment but this data as not available in the SEER data set. It is also possible that men over 70 are rarely screened and cases of screen-detected prostate cancer in this group are few.

There is increasing interest in the role of non cell autonomous factors in cancer progression and metastases and release from dormancy (or senescence) [11]. In this paradigm, host factors (macro-environment) may change over time and lead to a more permissive cancer niche (primary or metastatic) or may reactivate the growth of dormant cancer cells within the metastatic niche. Increasing co-morbidity with age leads to a higher likelihood death from prostate cancer and as men age there may be less resistance to keeping metastatic disease at bay through immune surveillance or other mechanisms. In a large study from Sweden, patient co-morbidity, as determined by the Charlson comorbidity index, was an independent predictor of crude prostate cancer mortality, but after adjustment for tumour characteristics and treatments, comorbidity was no longer a predictor of prostate cancer mortality [17].

Up to 30% of men at age 50 have histologic evidence of low-grade prostate cancer on post-mortem examination [18], yet death from prostate cancer is rare in young men and most deaths from prostate cancer occur at a much older age. The concept of tumor dormancy may help to explain why some prostate cancer deaths occur 25 years or more after diagnosis. It may be that aging is accompanied by a decline in innate resistance which leads to emergence from dormancy—either due to waning immunity, frailty or another mechanism (the frailty model is akin to the natural history of herpes zoster, which infects the nervous system early in life as chicken pox but usually does not manifest until late in life in the form of shingles).

We also document how time to death varies with Gleason grade. Among men over 60 initially diagnosed with low-grade cancers, we saw a continuous climb in the annual prostate cancer-specific mortality rate with time since diagnosis (Figure 3). In contrast, for men with high-grade cancers, the mortality rate declined with time since diagnosis (Figure 3) and the annual number of deaths from prostate cancer peaked early on (years one to three post-diagnosis) (Figure 4). Among those with high-grade cancers, 79.2% of prostate cancer deaths occurred within the first 10 years (Table 1).

There are several limitations of these data. We might have misclassified some high-grade patients as having Gleason ≤ 6 prostate cancer and those who died of prostate cancer actually had Gleason score ≥ 7 cancer. Since 1996, there have been two major updates to the Gleason scoring system [19,20] which have upgraded many of those historically considered to have Gleason ≤ 6 disease. We also know that historical biopsy techniques (e.g., 6-core biopsy) tended to under-stage patients. However, there is increasing evidence that tumour grade is not fixed but may be a dynamic process. Under this paradigm, Gleason ≤ 6 prostate cancer can progress into higher grade prostate cancers and this ultimately leads to cancer mortality [21,22,23,24,25,26]. Up to 30% of individuals initially diagnosed with Gleason 6 prostate cancer will ultimately be upstaged to higher grade disease. In a recent study of patients undergoing active surveillance Carter et al. reported that 40% of men with Gleason 6 prostate cancer were later upstaged to Gleason 7 or higher [26]. A study by Assel et al. concluded that grade progression was probable because the longer the time elapsed from an elevated PSA measurement to a diagnostic biopsy, the more likely was the lesion to be high grade [25].

It is also possible that Gleason ≤ 6 prostate cancer has the potential to metastasize from the outset and ultimately lead to patient mortality. This interpretation has some precedent from our work on early stage breast cancers [27]. We proposed that metastases is an early event among low grade breast tumors and that they undergo a long period of tumor dormancy prior to reactivation.

This increase in prostate cancer diagnosis may contribute to a lead-time bias, however, we do not have information on the method of diagnosis (screen-detected versus clinical) or treatments received for our cohort. A number of cancers may have been detected incidentally at the time of transurethral resection of the prostate for benign disease. Concerns have been raised regarding the accuracy of the PSA data within the SEER system [28] and therefore we chose not to include PSA data in our study. Further, by studying a large number of patients diagnosed over a short period of time we minimize potential confounding due to trends in screening intensity, diagnostic approaches and treatment.

There are several other limitations to our study. The time-period of diagnosis was selected to ensure that we had 25 years of follow-up data. This time period also saw a significant increase in the incidence of prostate cancer with the widespread adoption of prostate specific antigen (PSA) testing in the United States and changes in surgical norms [29,30,31]. The treatment cancer has changed in the United States since 1997 including the more widespread use of chemotherapy [32]. The data set is entirely from the United States and it would be of interest to see if similar patterns of mortality are seen in other countries such as those in Europe and the UK. It may not reflect prostate cancer case-fatality in other countries such as those in Africa. It will be of interest to revisit this cohort and to study new cohorts in the future to see if the pattern of mortality changes.

We have also chosen to present the data using annual mortality rates because these are more informative than traditional Kaplan–Meier curves for looking at changing rates over time and for addressing questions about tumour progression. Nevertheless, we have included the Kaplan–Meier survival curves for comparison purposes and for completeness (Figures S1–S4).

## 5. Conclusions

It is widely known that the incidence of and mortality from prostate cancer increases with age and prostate cancer is the second leading cause of death from cancer for men in their eighties [2]. To some extent the increase in mortality with age reflects the rising incidence rates but comorbidity plays and important role as well. We show here that 20 year prostate cancer survival rates decline with advancing age, in particular for men who present with non-metastatic low grade cancers. Further, for men diagnosed with Gleason score ≤ 6 prostate cancer, the annual prostate cancer mortality rate continued to rise 20 years after diagnosis and peaked after age 85. A man diagnosed with prostate cancer at age 80 is more likely to die of prostate cancer than a man diagnosed at age 60. As a result, a high proportion of prostate cancer deaths in men diagnosed from 1992 to 1997 in the USA occurred in men initially diagnosed with low-grade disease. Further, we show that attained age is a risk factor for prostate cancer mortality and is independent of age at diagnosis. As men age, the annual risk of death from prostate cancer increases. This is not seen for other cancer sites (for most cancer sites ten year survival is a good surrogate for cure). The reason for the rising death rate with age is not clear, but waning immunity, the contribution of comorbidity and the influences of other host factors are likely to be important and should be a topic of future study.

## Figures and Tables

**Table 1:** Demographic information for entire cohort.

Group	Frequency (%)	Frequency Percent of Category	Number of Deaths from Prostate Cancer (%)	Annual All-Cause Mortality Rate (%)	Annual Prostate Specific Mortality Rate (%)	10-Year Prostate Cancer Specific Survival Rate	20-Year Prostate Cancer Specific Survival Rate	Median Years to Prostate Specific Death (IQR)	% of Prostate Cancer Deaths within 10 Years of Diagnosis	% of Prostate Cancer Deaths between 10 and 20 Years of Diagnosis
**Overall**	116,796		21,896 (18.7%)	6.5%	1.5%	84.6%	74.5%	6.5 (2.5–12)	69.3%	25.6%
	**Age**									
<60	16,930	14.4%	2622 (15.4%)	2.3%	0.9%	90.2%	83.7%	7.5 (2.9–10)	60.3%	33.1%
60–70	43,566	37.3%	7371 (16.9%)	5.2%	1.2%	88.5%	78.7%	7.8 (3.3–13.6)	60.5%	33.6%
70+	56,300	48.2%	11,903 (21.1%)	11.7%	2.1%	78.7%	63.9%	4.8 (1.9–9.5)	76.7%	19.1%
	**Ethnicity**									
White	94,823	81.1%	17,185 (18.1%)	6.4%	1.4%	85.4%	75.6%	6.2 (2.5–11.6)	68.6%	26.2%
Black	14,545	12.4%	3469 (23.8%)	7.1%	1.9%	79.6%	67.3%	5.5 (2.1–10.9)	72.1%	23.3%
Other ^1^	6317	5.4%	1138 (18.0%)	6.5%	1.4%	85.0%	74.8%	6.0 (2.3–11.4)	69.5%	25.9%
Unknown	1111	0.9%	104 (9.3%)	6.1%	1.1%	87.2%	82.5%	4.3 (2.0–7.4)	83.6%	15.4%
	**Gleason score**									
≤6	81,056	69.3%	10,020 (12.3%)	5.8%	0.9%	91.1%	82.2%	8.3 (4.2–13.4)	59%	36.1%
≥7	23,285	19.1%	7995 (34.3%)	8.5%	3.1%	67.1%	52.6%	4.4 (1.9–8.8)	79.2%	19%
Missing	775	0.6%	330 (42.5%)	10.7%	5.5%	55.7%	44.4%	2.7 (1.2–6.8)	86.9%	11.2%
Unknown	11,680	10%	3551 (30.4%)	10.0%	3.4%	72.4%	62.2%	3.7 (1.2–10.4)	56.4%	30.6%
	**Metastases at Diagnosis**									
No metastases	94,934	81.2%	13,468 (14.1%)	6.0%	1.1%	89.5%	79.5%	8.2 (4.3–13.2)	60.5%	35%
Metastases present	7416	6.3%	4822 (65.0%)	17.8%	9.1%	22.1%	13.2%	1.8 (0.9–3.8)	95.6%	4.1%
Unknown	14,446	12.3%	3606 (24.9%)	7.8%	2.0%	78.2%	65.6%	6.0 (2.5–12.5)	66.9%	19.8%
	**Treatment**									
No Surgery	60,148 (51.4%)	51.4%	13,650 (22.6%)	9.2%	2.1%	79.1%	63.8%	5.3 (6.1–15.6)	51.2%	46.4%
Radical Prostatectomy	38,885 (33.2%)	33.2%	4112 (10.5%)	3.6%	0.6%	94.4%	87.6%	10.4 (6.1–15.6)	47.7%	44.3%
Other surgery ^2^	17,763 (15.2%)	15.2%	4134 (23.2%)	8.7%	1.8%	77.3%	68.6%	4.3 (1.8–9.5)	76.1%	13.1%

^1^ Includes individuals who identify as American Indian, Alaskan native and Asian heritage; ^2^ Includes individuals who underwent unknown surgery, TURP or Cryotherapy, subtotal/simple prostatectomy and cystoprostatectomy.

## Data Availability

The data presented in this study are available through the SEER registry.

## Supplementary Materials

The following supporting information can be downloaded at: https://www.mdpi.com/article/10.3390/cancers14174149/s1, Figure S1: Actuarial prostate cancer survival curve, by age at diagnosis; Figure S2: Actuarial prostate cancer survival curve, by Gleason score; Figure S3: Actuarial prostate cancer survival, by race; Figure S4: Actuarial prostate cancer survival curve, by metastatic status at presentation; Figure S5: Percentage of all deaths of prostate cancer by year of follow-up, by age at diagnosis; Figure S6: Percentage of all deaths of prostate cancer by year of follow-up, by Gleason score.

## References

[1] Epstein M.M., Edgren G., Rider J.R., Mucci L.A., Adami H.-O. (2012). Temporal trends in cause of death among Swedish and US men with prostate cancer. J. Natl. Cancer Inst..

[2] Miller K.D., Nogueira L., Devasia T., Mariotto A.B., Yabroff K.R., Jemal A., Kramer J., Siegel R.L. (2022). Cancer treatment and survivorship statistics, 2022. CA Cancer J. Clin..

[3] Riihimäki M., Thomsen H., Brandt A., Sundquist J., Hemminki K. (2011). What Do Prostate Cancer Patients Die of?. Oncologist.

[4] Milonas D., Ruzgas T., Venclovas Z., Jonusaite D., Matijosaitis A.J., Trumbeckas D., Varpiotas E., Auskalnis S., Skaudickas D., Mickevicius R. (2022). Effect of Clinical Parameters on Risk of Death from Cancer after Radical Prostatectomy in Men with Localized and Locally Advanced Prostate Cancer. Cancers.

[5] USPTF: Final Recommendation Statement: Prostate Cancer: Screening—US Preventive Services Task Force. https://www.uspreventiveservicestaskforce.org/Page/Document/RecommendationStatementFinal/prostate-cancer-screening.

[6] Rawla P. (2019). Epidemiology of Prostate Cancer. World J. Oncol..

[7] Bechis S.K., Carroll P.R., Cooperberg M.R. (2011). Impact of age at diagnosis on prostate cancer treatment and survival. J. Clin. Oncol..

[8] Bernard B., Burnett C., Sweeney C.J., Rider J.R., Sridhar S.S. (2020). Impact of age at diagnosis of de novo metastatic prostate cancer on survival. Cancer.

[9] Pettersson A., Robinson D., Garmo H., Holmberg L., Stattin P. (2018). Age at diagnosis and prostate cancer treatment and prognosis: A population-based cohort study. Ann. Oncol..

[10] Hall W.H., Jani A.B., Ryu J.K., Narayan S., Vijayakumar S. (2005). The impact of age and comorbidity on survival outcomes and treatment patterns in prostate cancer. Prostate Cancer Prostatic Dis..

[11] Feunteun J., Ostyn P., Delaloge S. (2022). Tumor cell malignancy: A complex trait built through reciprocal interactions between tumors and tissue-body system. iScience.

[12] Kaplan E.L., Meier P. (1958). Nonparametric Estimation from Incomplete Observations. J. Am. Stat. Assoc..

[13] D’Arrigo G., Leonardis D., Abd ElHafeez S., Fusaro M., Tripepi G., Roumeliotis S. (2021). Methods to Analyse Time-to-Event Data: The Kaplan-Meier Survival Curve. Oxidative Med. Cell. Longev..

[14] Narod S.A., Giannakeas V., Sopik V. (2018). Time to death in breast cancer patients as an indicator of treatment response. Breast Cancer Res. Treat..

[15] SEER Incidence Database—SEER Data & Software. https://seer.cancer.gov/data/index.html.

[16] von Elm E., Altman D.G., Egger M., Pocock S.J., Gøtzsche P.C., Vandenbroucke J.P., STROBE Initiative (2007). Strengthening the Reporting of Observational Studies in Epidemiology (STROBE) statement: Guidelines for reporting observational studies. BMJ.

[17] Rajan P., Sooriakumaran P., Nyberg T., Akre O., Carlsson S., Egevad L., Steineck G., Wiklund N.P. (2017). Effect of Comorbidity on Prostate Cancer-Specific Mortality: A Prospective Observational Study. J. Clin. Oncol..

[18] Zlotta A.R., Egawa S., Pushkar D., Govorov A., Kimura T., Kido M., Takahashi H., Kuk C., Kovylina M., Aldaoud N. (2013). Prevalence of prostate cancer on autopsy: Cross-sectional study on unscreened Caucasian and Asian men. J. Natl. Cancer Inst..

[19] Epstein J.I., Allsbrook W.C., Amin M.B., Egevad L.L., ISUP Grading Committee (2005). The 2005 International Society of Urological Pathology (ISUP) Consensus Conference on Gleason Grading of Prostatic Carcinoma. Am. J. Surg. Pathol..

[20] Epstein J.I., Egevad L., Amin M.B., Delahunt B., Srigley J.R., Humphrey P.A., Grading Committee (2016). The 2014 International Society of Urological Pathology (ISUP) Consensus Conference on Gleason Grading of Prostatic Carcinoma: Definition of Grading Patterns and Proposal for a New Grading System. Am. J. Surg. Pathol..

[21] Jiang Y., Meyers T.J., Emeka A.A., Cooley L.F., Cooper P.R., Lancki N., Helenowski I., Kachuri L., Lin D.W., Stanford J.L. (2022). Genetic Factors Associated with Prostate Cancer Conversion from Active Surveillance to Treatment. HGG Adv..

[22] Godtman R.A., Kollberg K.S., Pihl C.G., Månsson M., Hugosson J. (2022). The Association Between Age, Prostate Cancer Risk, and Higher Gleason Score in a Long-term Screening Program: Results from the Göteborg-1 Prostate Cancer Screening Trial. Eur. Urol..

[23] Frånlund M., Månsson M., Godtman R.A., Aus G., Holmberg E., Kollberg K.S., Lodding P., Pihl C.G., Stranne J., Lilja H. (2022). Results from 22 years of Follow-up in the Göteborg Randomized Population-Based Prostate Cancer Screening Trial. J. Urol..

[24] Richard P.O., Timilshina N., Komisarenko M., Martin L., Ahmad A., Alibhai S.M.H., Hamilton R.J., Kulkarni G.S., Finelli A. (2020). The long-term outcomes of Gleason grade groups 2 and 3 prostate cancer managed by active surveillance: Results from a large, population-based cohort. Can. Urol. Assoc. J..

[25] Assel M., Dahlin A., Ulmert D., Bergh A., Stattin P., Lilja H., Vickers A.J. (2018). Association Between Lead Time and Prostate Cancer Grade: Evidence of Grade Progression from Long-term Follow-up of Large Population-based Cohorts Not Subject to Prostate-specific Antigen Screening. Eur. Urol..

[26] Carter H.B., Helfand B., Mamawala M., Wu Y., Landis P., Yu H., Wiley K., Na R., Shi Z., Petkewicz J. (2019). Germline Mutations in ATM and BRCA1/2 Are Associated with Grade Reclassification in Men on Active Surveillance for Prostate Cancer. Eur. Urol..

[27] Giannakeas V., Sopik V., Narod S.A. (2020). Association of a Diagnosis of Ductal Carcinoma In Situ With Death From Breast Cancer. JAMA Netw. Open..

[28] PSA Values and SEER Data. https://seer.cancer.gov/data/psa-values.html.

[29] Welch H.G., Kramer B.S., Black W.C. (2019). Epidemiologic Signatures in Cancer. N. Engl. J. Med..

[30] Negoita S., Mariotto A., Benard V., Kohler B.A., Jemal A., Penberth (2019). Reply to annual report to the nation on the status of cancer, part II: Recent changes in prostate cancer trends and disease characteristics. Cancer.

[31] Lu-Yao G.L., McLerran D., Wasson J., Wennberg J.E. (1993). An Assessment of Radical Prostatectomy: Time Trends, Geographic Variation, and Outcomes. JAMA.

[32] Fallara G., Robesti D., Nocera L., Raggi D., Marandino L., Belladelli F., Montorsi F., Malavaud B., Ploussard G., Necchi A. (2022). Chemotherapy and advanced androgen blockage, alone or combined, for metastatic hormone-sensitive prostate cancer a systematic review and meta-analysis. Cancer Treat Rev..

