# Supplementary material for: The Effect of Age on Prostate Cancer Survival
Source: Cancers (Basel). 2022 Aug 27;14(17):4149. doi: 10.3390/cancers14174149 (PMC9454626; doi:10.3390/cancers14174149)

Supplementary for Manuscript Entitled

The Effect of Age on Prostate Cancer Survival

Figure S1. Actuarial prostate cancer survival curve, by age at diagnosis....................................... 2

Figure S2. Actuarial prostate cancer survival curve, by Gleason score.. 3

Figure S3. Actuarial prostate cancer survival, by race 4

Figure S4. Actuarial prostate cancer survival curve, by metastatic status at presentation 5

Figure S5. Percentage of all deaths of prostate cancer by year of follow-up, by age at diagnosis. 6

Figure S6. Percentage of all deaths of prostate cancer by year of follow-up, by Gleason score.... 7

Figure S1. Actuarial prostate cancer survival curve, by age at diagnosis

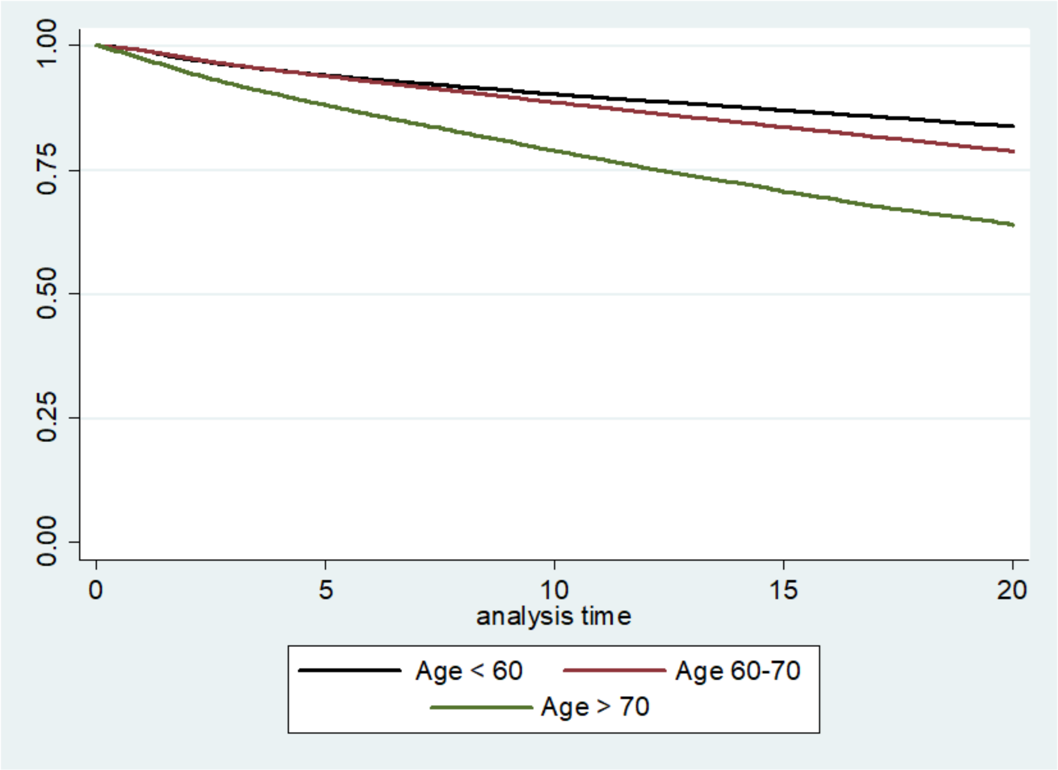

Figure S2. Actuarial prostate cancer survival curve, by Gleason score

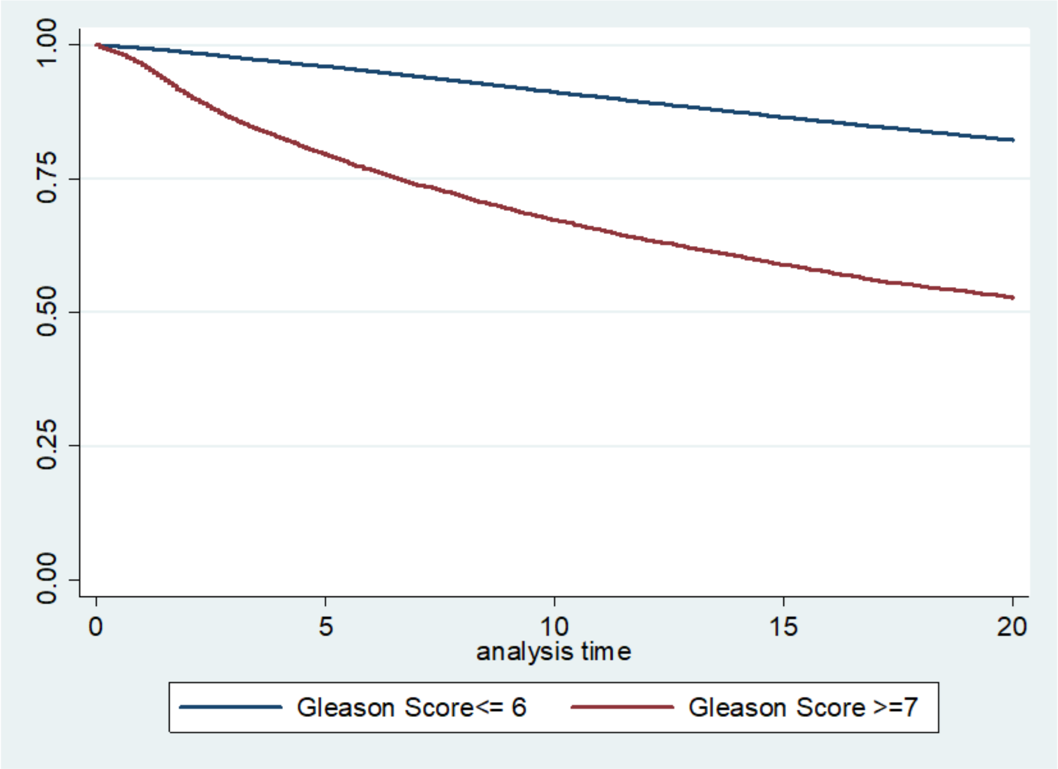

Figure S3. Actuarial prostate cancer survival, by race

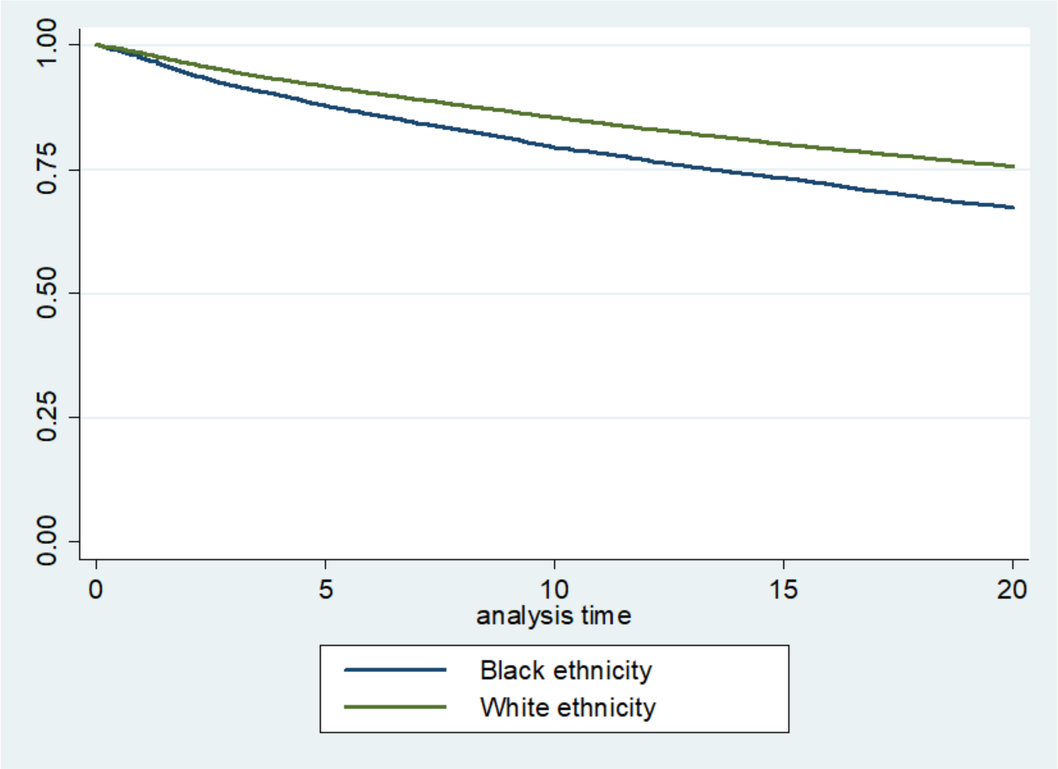

Figure S4. Actuarial prostate cancer survival curve, by metastatic status at presentation

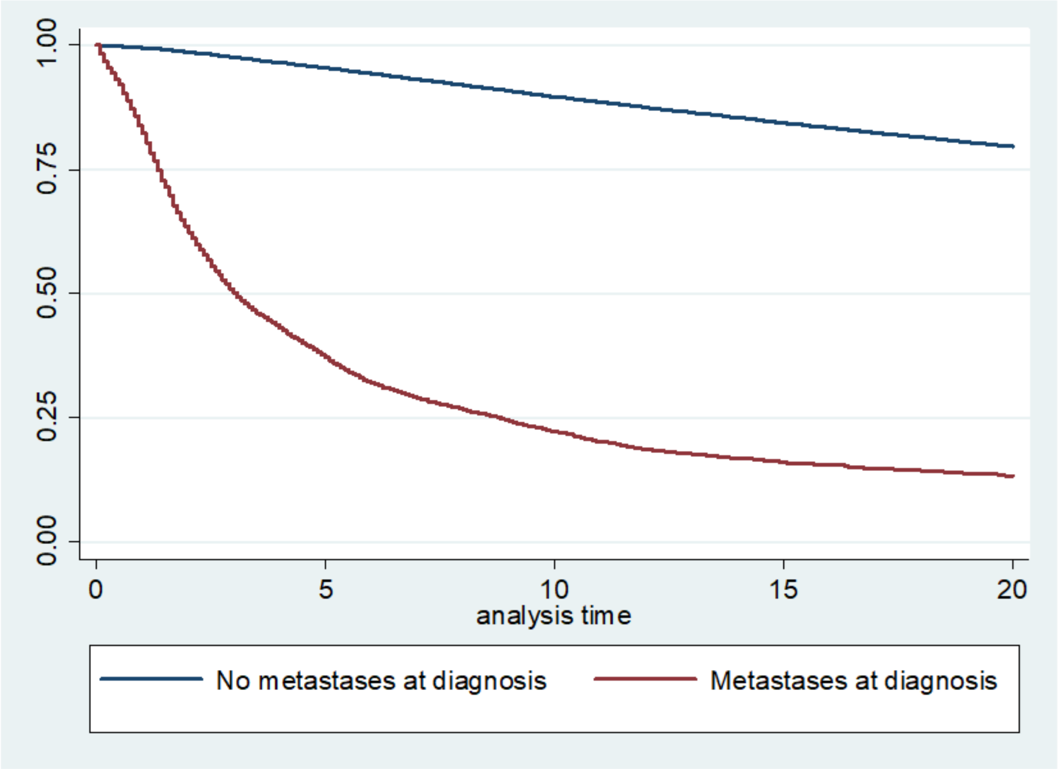

Figure S5. Percentage of all deaths of prostate cancer by year of follow-up, by age at diagnosis

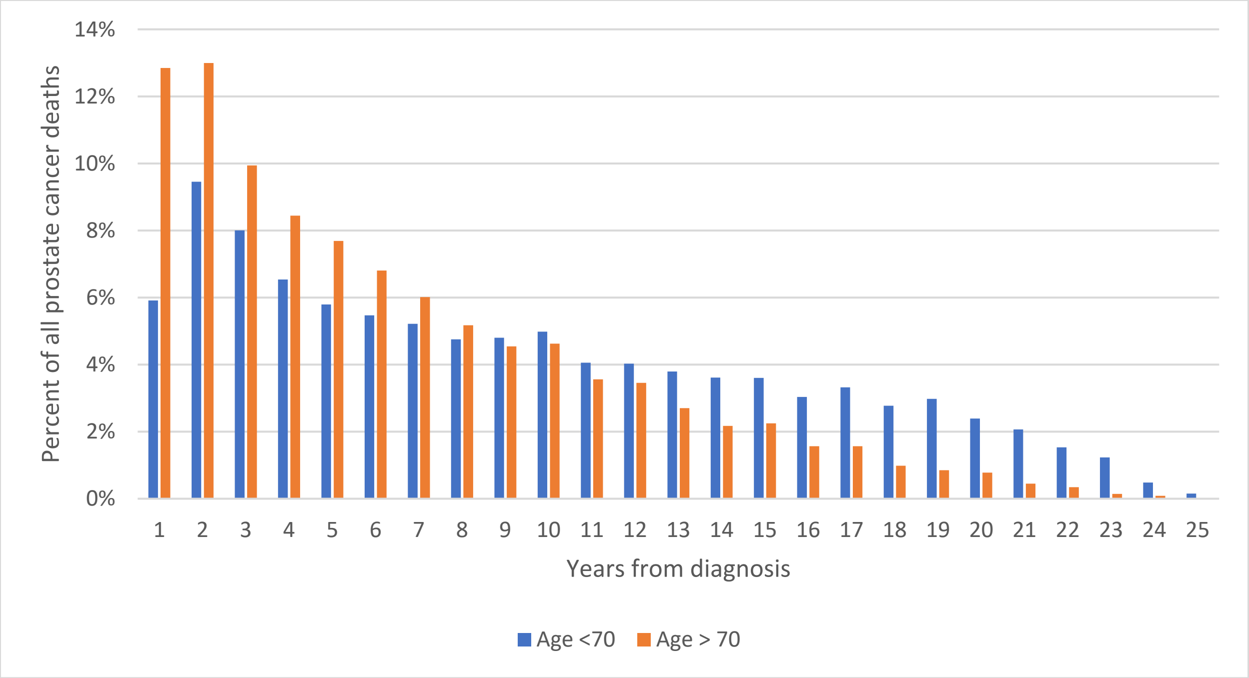

Figure S6. Percentage of all deaths of prostate cancer by year of follow-up, by Gleason score

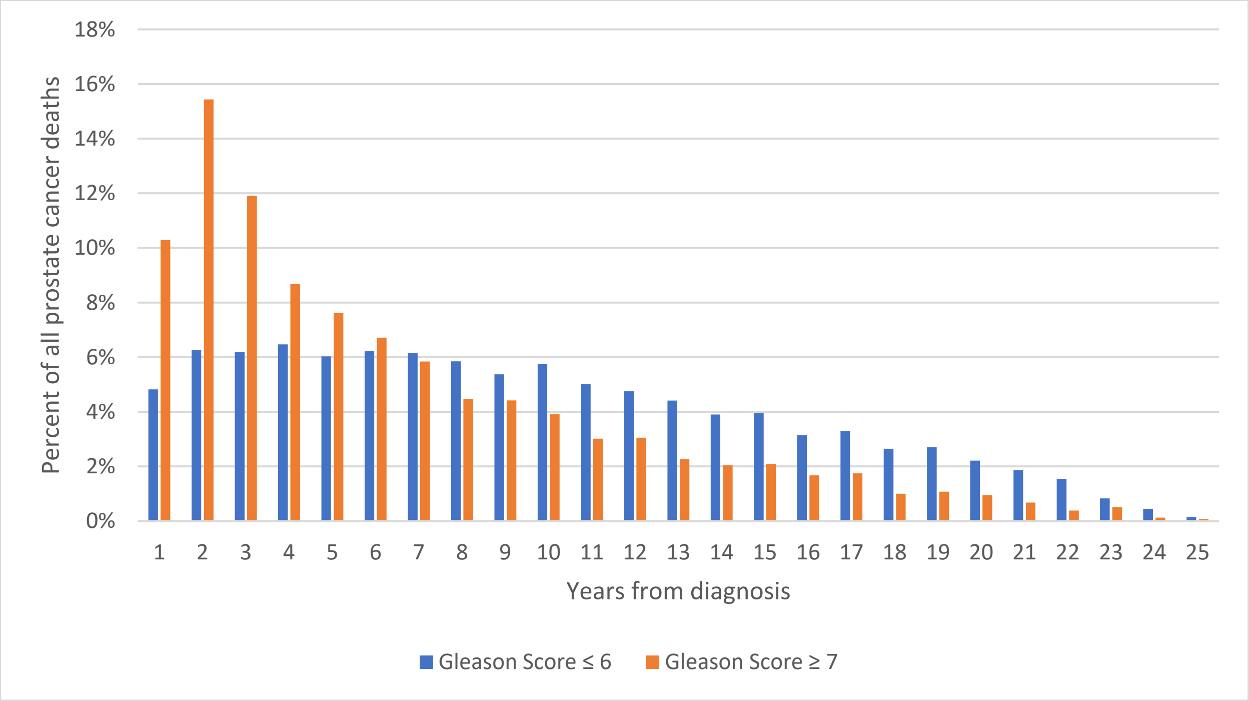

Supplement: Supplementary file 1 [file cancers-14-04149-s001.zip › cancers-1789618-supplementary.pdf]
